# Genomic landscape of pleural mesothelioma in Japanese patients: A comprehensive analysis using nationwide database

**DOI:** 10.1016/j.tranon.2026.102731

**Published:** 2026-03-18

**Authors:** Hirokazu Taniguchi, Kazumasa Akagi, Takahito Fukuda, Takuya Honda, Hirokazu Kurohama, Nozomi Ueki, Yuki Matsuoka, Emiko Udo, Saya Yahata, Shoko Miura, Hiromi Tomono, Noritaka Honda, Yosuke Dotsu, Midori Matsuo, Shinnosuke Takemoto, Izumi Sato, Shinji Okano, Masahiro Nakashima, Hiroshi Mukae, Kazuto Ashizawa

**Affiliations:** a Clinical Oncology Center, Nagasaki University Hospital, Nagasaki, Japan; b Department of Respiratory Medicine, Nagasaki University Graduate School of Biomedical Sciences, Nagasaki, Japan; c Department of Gastroenterology and Hepatology, Nagasaki University Graduate School of Biomedical Sciences, Nagasaki, Japan; d Department of Tumor and Diagnostic Pathology, Atomic Bomb Disease Institute, Nagasaki University, Nagasaki, Japan; e Cancer Genomics Unit, Clinical Genomics Center, Nagasaki University Hospital, Nagasaki, Japan; f Department of Obstetrics and Gynecology, Nagasaki University Graduate School of Biomedical Sciences, Nagasaki, Japan; g Clinical Research Center, Nagasaki University Hospital, Nagasaki, Japan; h Department of Clinical Epidemiology, Graduate School of Biomedical Sciences, Nagasaki University, Nagasaki, Japan; i Department of Pathology, Nagasaki University Graduate School of Biomedical Sciences, Nagasaki, Japan; j Department of Clinical Oncology, Nagasaki University Graduate School of Biomedical Sciences, Nagasaki, Japan

**Keywords:** Mesothelioma, Comprehensive cancer genomic profiling, Gene alterations, Database analysis, Cancer genomic medicine

## Abstract

Pleural mesothelioma (PM) is a rare and aggressive malignancy. The development of novel therapeutic strategies targeting PM remains an unmet clinical need. However, comprehensive genomic data from Asian populations, particularly from Japanese patients, are limited. This study aimed to elucidate the genomic landscape of PM in Japanese patients using the nationwide Center for Cancer Genomics and Advanced Therapeutics (C-CAT) genomic database. A total of 211 patients registered between June 2019 and March 2025 were analyzed. The most frequent genetic alterations were in *BAP1, NF2, TP53, CDKN2A/B*, and *MTAP*. The median tumor mutation burden (TMB) was 1.26, and no microsatellite instability–high patients were detected. The median overall survival (OS) after first-line treatment was 30.6 months. Patients treated with immune checkpoint inhibitors (ICIs) had a significantly better OS than those who did not receive ICIs. In univariate and multivariate analyses, *TP53* alterations and high TMB (cutoff value of 1.6) were associated with poor prognosis. These results suggest that integrating clinical and genomic data can enhance prognostic stratification and contribute to the development of precision medicine for PM. This study provides the first large-scale genomic characterization of Japanese PM patients with C-CAT and highlights potential biomarkers for future therapeutic development.

## Highlights

• Large-scale genomic profiling of Japanese patients with pleural mesothelioma.
• Frequent alterations identified in BAP1, NF2, TP53, CDKN2A/B, and MTAP.
• High TMB and TP53 alterations were associated with poor prognosis.
• Immune checkpoint inhibitor treatment improved overall survival.

## Introduction

Pleural mesothelioma (PM) is a rare and aggressive malignancy that primarily arises from asbestos exposure. Because of the long latency period (typically 30–50 years) between asbestos exposure and disease onset, the incidence of PM is expected to continue increasing, even in countries where asbestos use has been banned [1]. For unresectable PM, several systemic therapies have been established as 1^st^ line standard treatment. The combination of immune checkpoint inhibitors (ICIs), nivolumab and ipilimumab, demonstrated a significant survival benefit in the phase III CheckMate 743 trial, underscoring that immunotherapy conferred a significant survival benefit as compared to platinum-pemetrexed chemotherapy [2]. Cytotoxic chemotherapy with cisplatin plus pemetrexed [3] and, more recently, the triplet regimen of cisplatin, pemetrexed, and pembrolizumab demonstrated efficacy in a phase III KEYNOTE-483 trial [4]. Nevertheless, because PM is a rare cancer, the implementation of large-scale clinical trials remains challenging, and the development of new therapeutic agents has lagged behind that for more common malignancies.

Genetically, PM is characterized by frequent mutations and copy number alterations in *BAP1, CDKN2A*, and *NF2*, including both mutations and copy number alterations [5]. These genes function as tumor suppressors, making them difficult to target them directly using current molecular therapies. Comprehensive genomic analyses, such as those from The Cancer Genome Atlas (TCGA), which included 74 PM patients [6], and single-cell RNA sequencing studies [7], TCGA and the NCI cohort, which included 113 mesothelioma patients (99 pleural and 14 peritoneal mesothelioma), have provided valuable insights into the genomic landscape of PM. However, in non-small-cell lung cancer, *EGFR* mutations are more common in Asians, whereas *KRAS* mutations are more frequent in Western populations, indicating that there may be racial differences in oncogene-related information [8]. In previous reports on the genomic landscape of PM, only a few Asian patients were included. Consequently, genomic data on PM in Asian populations, particularly Japanese populations, remain extremely limited.

Cancer genomic medicine (CGM) aims to optimize therapeutic strategies based on the genomic profile of each tumor, and has demonstrated clinical utility across a wide range of malignancies [[9], [10], [11]]. In Japan, comprehensive genomic profiling (CGP) tests were approved for reimbursement in 2019, allowing their implementation in clinical practice for patients with rare cancers or those who have completed standard therapy. Center for Cancer Genomics and Advanced Therapeutics (C-CAT) was established to systematically collect and utilize genomic and clinical data from CGP tests. The C-CAT aggregates genomic and clinical information across Japan, and its database is accessible to hospitals, academic institutions, and the pharmaceutical industry for research and development purposes [12]. The C-CAT registry includes detailed clinical parameters, such as patient demographics, family history, prior malignancies, therapeutic history, and outcomes, enabling integrated analyses of genomic and real-world clinical data.

In this study, we aimed to characterize the genomic alterations and clinical features of PM in Japanese patients registered in the C-CAT. Furthermore, to explore the potential ethnic differences in the molecular landscape of PM, we performed comparative analyses using publicly available datasets, including TCGA.

## Material and methods

### Study design and data source

This retrospective, nationwide cohort study used data from the C-CAT database in Japan. The C-CAT database comprises clinical and genomic data, such as demographics, prior malignancies, treatment history, and outcomes derived from CGP tests conducted at CGM-designated hospitals. Based on this data, C-CAT generates standardized reports that include clinical annotations and information on matched clinical trials. In Japan, several CGP tests have been approved and are currently available, including the FoundationOne CDx and FoundationOne Liquid CDx (Foundation Medicine Inc., Cambridge, MA, USA), OncoGuide NCC Oncopanel System (Sysmex Co., Ltd., Kobe, Japan), Guardant360 CDx (Guardant Health Inc., Palo Alto, CA, USA), and GenMine TOP (Konica Minolta, Inc., Tokyo, Japan,Table S1). Primary analysis of the genomic data was conducted according to the protocols defined by each CGP test manufacturer. As each testing company employs its own file format to report genomic data, C-CAT has established a standardized format. Testing companies are required to submit genomic data in a unified format to ensure consistency and interoperability across datasets.

### Study patients and data items/definitions

Patients with pleural mesothelioma who underwent CGP between June 2019 and March 2025 were included in this study. Through the C-CAT database, database queries were conducted by navigating to the Cancer Type section, selecting "Pleura (PLEURA)" as the first-level category and "Pleural Mesothelioma" as the second-level category. All patients flagged within the designated research period (June 2019 to March 2025) were selected, and both case- and report-level CSV files were downloaded. We used the following variables for analysis: clinical variables, including age, sex, Eastern Cooperative Oncology Group performance status (PS), smoking history, histological classification, CGP test type, and treatment regimen, and genomic variables, including single-nucleotide variants, copy number alterations, rearrangements (in DNA and RNA), and biomarkers such as tumor mutation burden and microsatellite instability. For histological classification, the records were reviewed to confirm that each patient had a unique histological designation. When both the "unclassified" and other histological types were present, the non-unclassified histology was adopted. If multiple conflicting histologic types were listed, the "unclassified" histology was adopted. Similarly, for the therapeutic agents, all cells containing pre- and post-expert panel treatment information were extracted for each case and reviewed to confirm that each patient had a unique regimen. Any obvious errors in the drug names or treatment indications were corrected individually. Patients were considered to have received ICIs if they were treated with nivolumab, ipilimumab, atezolizumab, or pembrolizumab as primary or adjuvant therapy. Patients were considered alteration-positive if abnormalities were detected in single-nucleotide variants, copy number alterations, or gene rearrangements.

### Statistical analysis

We summarized patients’ baseline characteristics using descriptive statistics. The primary endpoint was overall survival (OS), which was defined as the time from the initiation of systemic therapy; for recurrent cases, OS was calculated from the start date of systemic therapy excluding adjuvant treatment. We estimated OS using the Kaplan–Meier method and assessed survival differences between the following groups using the log-rank test: ICI treatment (yes vs no), histologic subtype (epithelioid vs. non-epithelioid), tumor mutation burden (TMB-high vs. TMB-low), and major gene alterations (*TP53, BAP1, CDKN2A,* etc.). Gene alterations observed in less than 10 patients were excluded from the analysis. We determined the optimal cutoff value for TMB to stratify survival outcomes using the Surv _cutpoint function from the Survminer package. We performed univariate and multivariate Cox proportional hazards regression analyses to identify prognostic factors for OS. We selected covariates based on both statistical significance in univariate analysis and their previously established prognostic relevance in the literature. We assessed the proportional hazard assumption using Schoenfeld residuals and confirmed that no violations were observed. We confirmed that all variance inflation factors for the covariates in the final model were below 2, indicating no multicollinearity. For external comparison, somatic mutation data categorized under "Simple Nucleotide Variation" were retrieved from the TCGA-MESO project using the TCGAbiolinks package (version 2.36.0) in R. Following data acquisition, a mutation annotation format object was constructed and processed. Subsequently, the top 50 genes ranked by mutation frequency were identified and an oncoplot was generated to visualize the mutational landscape. Analyses were performed using R software (version 4.5.0), and all tests were two-sided with a significance threshold of p < 0.05.

## Results

### Patients’ characteristics

The patient characteristics are summarized in Table 1. Between June 2019 and March 2025, 211 patients who provided informed consent and were registered in the C-CAT database were included in the study. This study included 151 males and 60 females with a median age of 68 years. The PS was 0/1/2/3/4 in 73/112/7/1/18 patients, respectively. In total, 135 patients had a history of smoking. Asbestos exposure history was not registered in the C-CAT database. Histologically, 119 patients were classified as epithelioid, 15 as biphasic, 24 as sarcomatoid, one as desmoplastic, and 52 as unknown. Eighty-three patients received ipilimumab plus nivolumab (IPI/NIVO) as first-line therapy and 186 patients received ICIs, including IPI/NIVO, at any point during their clinical course. The median number of prior treatments before CGP was 2 (range, 0–7). Regarding the types of CGP tests, 167 patients underwent FoundationOne CDx, 23 underwent FoundationOne Liquid, 15 underwent NCC Oncopanel, one underwent Guardant360, and five underwent GenMineTOP (Table 1). All cases were submitted from formalin-fixed, paraffin-embedded samples for tissue-based CGP. There are the guidelines for the handling of pathological tissue specimens for genomic medicine in Japan. And there were two cases in which no SNVs were detected; however, it is unclear whether this reflects technical failure or true biological findings.

**Table 1:** Patient characteristics.

Characteristic	N = 211^1^
**Age**	68.0 [59.0, 75.0]
**Sex**	
Male	151 (72%)
Female	60 (28%)
**ECOG PS**	
0	73 (35%)
1	112 (53%)
2	7 (3.3%)
3	1 (0.5%)
Unknown	18 (8.5%)
**Smoking history**	
Yes	135 (64%)
None	71 (34%)
Unknown	5 (2.4%)
**Histrogic type**	
Epithelioid Type	119 (56%)
Sarcomatoid Type	24 (11%)
Biphasic Type	15 (7.1%)
Desmoplastic Type	1 (0.5%)
Unknown	52 (25%)
**CGP type**	
FoundationOne CDx	167 (79%)
F1Liquid CDx	23 (11%)
NCC OncoPanel	15 (7.1%)
GenMineTOP	5 (2.4%)
Guardant360 CDx	1 (0.5%)
**First line treatment**	
NIVO + IPI	83 (39%)
Cisplatin + Pemetrexed	68 (32%)
Other	50 (24%)
Unknown	10 (4.7%)
**ICI use in all treatment**	
Yes	186 (88%)
None	15 (7.1%)
Unknown	10 (4.7%)

1 Median [Q1, Q3]; n (%)

### Identified genetic alterations

**Fig. 1:**
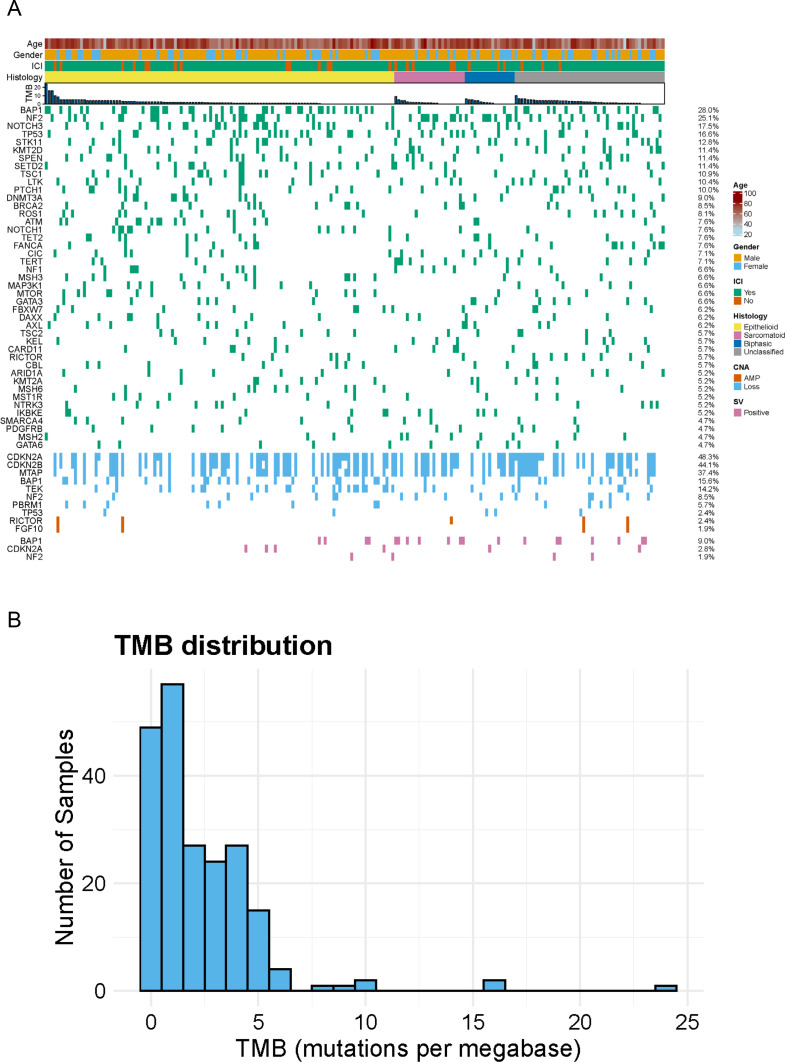
Genomic landscape of pleural mesothelioma in C-CAT. (A) The top row of the plot displays clinical information for each case, with tumor mutational burden represented as a bar plot. Single nucleotide variants (SNVs) and copy number alterations (CNAs) are arranged in descending order of frequency. For BAP1, CDKN2A, and NF2, patients are labeled as "SV positive" if any abnormality is detected in SNV, CNA, or gene rearrangement. The proportion of patients harboring each gene alteration is indicated to the right of each gene row, with the legend positioned further to the right. (B) The distribution of tumor mutation burden. The x-axis represents tumor mutation burden (mutations per megabase), while the y-axis indicates the number of samples. Abbreviations: ICI, immune checkpoint inhibitor; SNV, single nucleotide variant; CNA, copy number alteration; SV, structural variant; AMP, amplification; Mb, megabase.

The detected genetic alterations are shown in Fig. 1A. The most frequently observed gene mutations were *BAP1, NF2, NOTCH3, TP53*, and *STK11*. Copy number changes were observed in *CDKN2A, CDKN2B, MTAP, BAP1*, and *TEK* (Fig. 1A). The median TMB was 1.26 (range, 0–24,Fig. 1B). No microsatellite instability–high (MSI-H) patients were detected (data not shown). One patient harbored the rare fusion gene, *EWSR1–ATF*. Heterozygous germline loss-of-function variants in *BAP1* are associated with a high frequency of mesothelioma [13], however, there were no cases with germ line BAP1 alterations in this database.

When compared with data from the TCGA databases, which mainly include Western populations, the overall pattern of genetic alterations was largely consistent, particularly with the high prevalence of *BAP1, NF2*, and *TP53* alterations. Although the differences were not statistically significant, the spectrum of detected genes showed some variation compared to those reported in the databases (Fig. 1, Fig.. S1).

### Exploration of prognostic factors based on histologic subtype, therapeutic agents, and genetic alterations

**Fig. 2:**
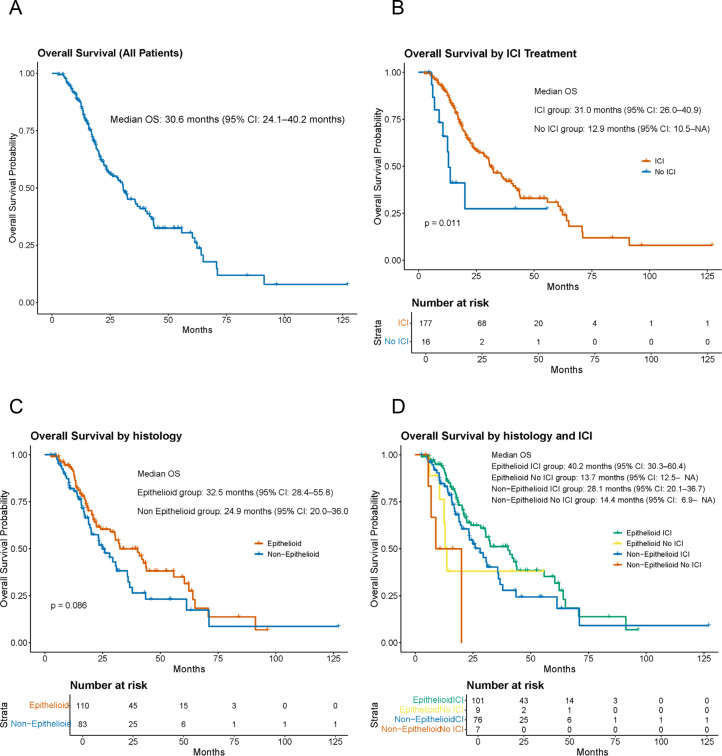
Overall survival based on clinical characteristics. (A) Overall survival of all pleural mesothelioma patients. Overall survival was calculated from the date of recurrence, excluding pre-operative and post-operative treatments. (B) Kaplan–Meier curves comparing overall survival between patients with and without immune checkpoint inhibitor (ICI) treatment. The p-value was established using the log-rank test. (C) Kaplan–Meier curves comparing overall survival between patients with epithelioid and non-epithelioid histology. The p-value was established using the log-rank test. (D) Kaplan–Meier curves comparing overall survival across four groups stratified by histological subtype and ICI treatment status. Abbreviations: ICI, immune checkpoint inhibitor.

In the C-CAT cohort, the median OS from the initiation of first-line therapy was 30.6 months (95% CI, 24.1–40.2,Fig. 2A). Among the pharmacological treatments for PM, ICIs represent key therapeutic options. When outcomes were analyzed according to ICI use, patients who received ICIs showed significantly better prognosis compared with those who did not (p = 0.012; 31.0 months [95% CI, 26.0–40.9] vs. 12.9 months [95% CI, 10.5–NA],Fig. 2B).

Survival analyses according to histologic subtype using the C-CAT dataset revealed that no statistically significant difference was observed between epithelioid and non-epithelioid subtypes; however, there was a trend toward longer OS in the epithelioid group (32.5 months [95% CI, 28.4–55.8] vs. 24.9 months [95% CI, 20.0–36.0],Fig. 2C). Both epithelioid and non-epithelioid subtypes showed improved outcomes with ICI treatment. However, because the number of patients who did not receive ICIs was limited, these results should be interpreted cautiously. Among the ICI-treated patients, those with epithelioid histology tended to have better survival (Fig. 2D). It should also be noted that in the Kaplan–Meier analysis stratified by detailed histologic classification, 46 of the 211 patients were categorized as “unclassified,” which may affect the interpretation of subtype-based OS comparisons (Table 1, Fig.. S2).

**Fig. 3:**
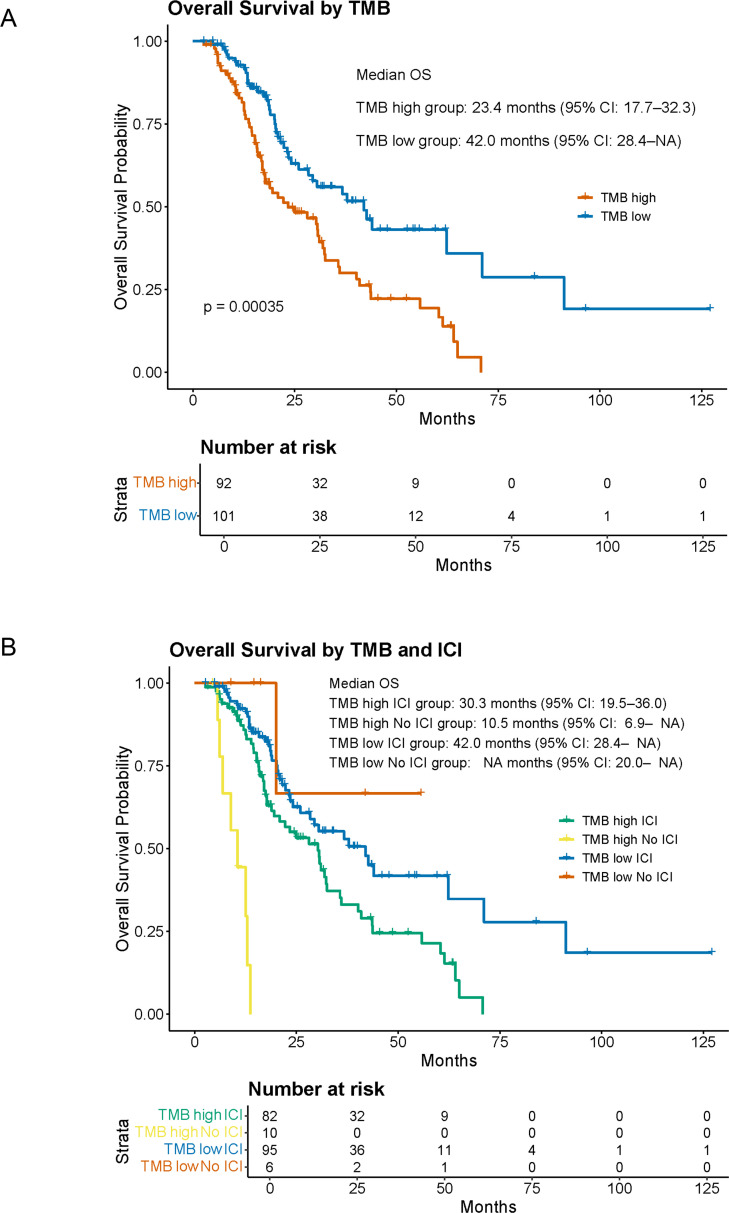
Overall survival analysis focusing on tumor mutation burden. (A) Kaplan–Meier curves comparing overall survival between patients with high and low TMB. TMB-high was defined as a value ≥ 1.6 mutations per megabase, and TMB-low as < 1.6. The p-value was established using the log-rank test. (B) Kaplan–Meier curves comparing overall survival across four groups stratified by TMB value and immune checkpoint inhibitor treatment status. Abbreviations: ICI, immune checkpoint inhibitor; TMB, tumor mutation burden.

TMB has been reported as a potential biomarker for predicting ICI efficacy. In other cancer types, patients with high TMB levels have been shown to exhibit better responses to ICI therapy [14]. In the present cohort, only five patients had a TMB of ≥10 mutations per megabase, and the median TMB was 1.26 (Fig. 1B). However, when patients were stratified into TMB-high and TMB-low groups using a cutoff value of 1.6, OS was significantly longer in the TMB-low group than in the TMB-high group (p = 0.00064; 42.0 months [95% CI, 28.4–NA] vs. 23.4 months [95% CI, 17.7–32.3],Fig. 3A). Furthermore, among patients with a high TMB, those who received ICIs showed an improved prognosis compared to those who did not (Fig. 3B). Previous reports have suggested that blood-based TMB tends to be higher than tissue-based TMB [15], therefore, caution may be warranted when interpreting tissue and liquid TMB together. In fact, when we analyzed our database, liquid-based TMB was significantly higher than tissue-based TMB (Fig. S3A). For this reason, we conducted separate analyses of TMB derived from tissue-based panels and liquid-based panels. Although the number of cases analyzed using liquid biopsy was limited (n = 20; cases analyzed with Guardant360 were excluded because TMB was not available), this additional analysis was performed. In both the tissue-panel and liquid-panel analyses, the TMB-high group showed a tendency toward poorer prognosis, which was consistent with the overall results (Fig. S3B).

**Fig. 4:**
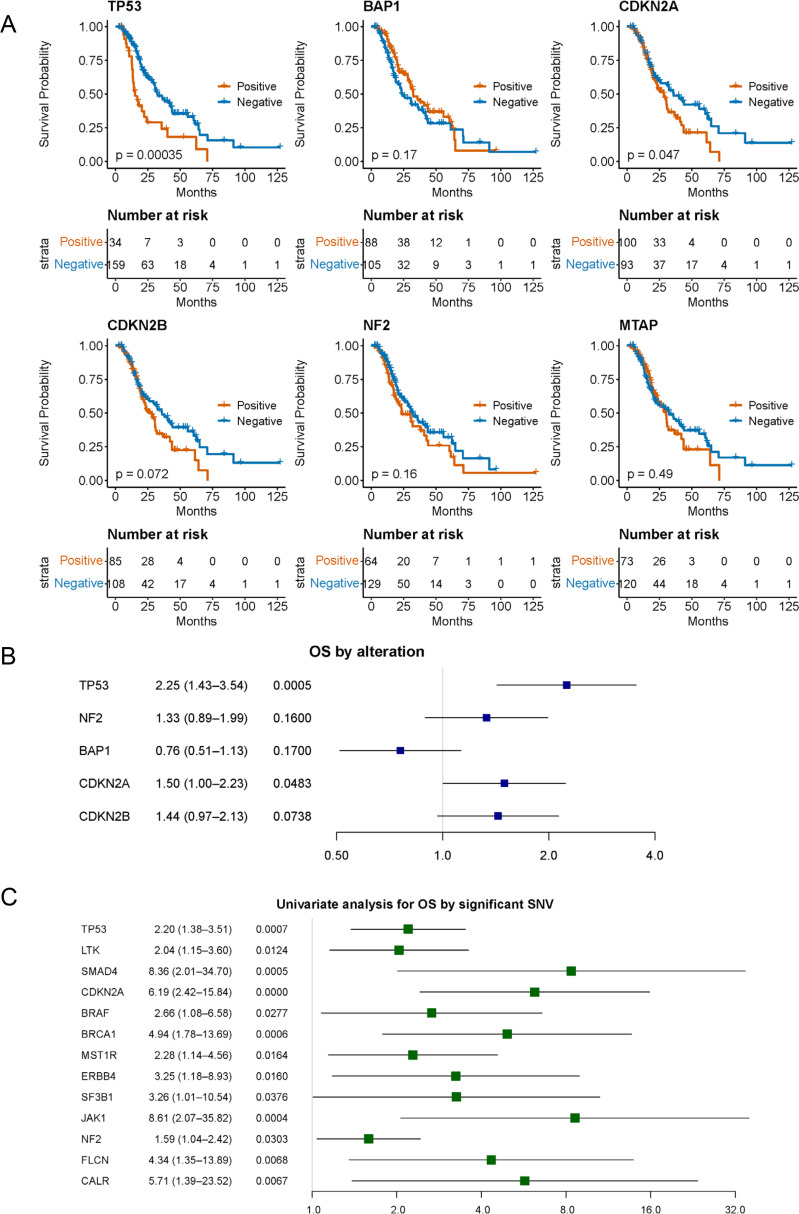
Overall survival analysis focusing on genomic alterations. (A) Kaplan–Meier analysis of overall survival stratified by the presence or absence of specific genomic alterations. The p-value was established using the log-rank test. (B) Forest plot showing hazard ratios from univariate Cox regression analysis of overall survival, stratified by the presence or absence of specific genomic alterations. The x-axis indicates hazard ratios, with values >1 suggesting worse prognosis. (C) Forest plot showing hazard ratios from univariate Cox regression analysis of overall survival, stratified by the presence or absence of single nucleotide variants. The x-axis indicates hazard ratios, with values >1 suggesting worse prognosis. Abbreviations: OS, overall survival, SNV, single nucleotide variant.

Next, we assessed the prognostic impact of the individual gene alterations. Among representative genomic alterations in PM, including *TP53, BAP1, NF2, CDKN2A, CDKN2B*, and *MTAP*, OS was shorter in patients harboring *TP53* mutations (15.2 months [95% CI, 13.4–35.7] vs. 32.3 months [95% CI, 29.4–43.7]) and *CDKN2A* alterations (28.4 months [95% CI, 22.3–36.7] vs. 36.0 months [95% CI, 24.1–62.3]) compared with those without these alterations (Fig. 4A, B). In contrast, alterations in *BAP1, NF2, CDKN2B*, and *MTAP* expression were not significantly associated with OS (Fig. 4A, B). *MTAP* loss is frequently associated with *CDKN2A* loss, therefore, we analyzed regarding the overlap between *CDKN2A* loss and *MTAP* loss, 79 cases harbored both alterations, whereas 23 cases had *CDKN2A* loss alone. Analysis of OS showed a tendency for an earlier decline in the Kaplan–Meier curve in cases with *CDKN2A* loss alone (Fig. S4).

**Fig. 5:**
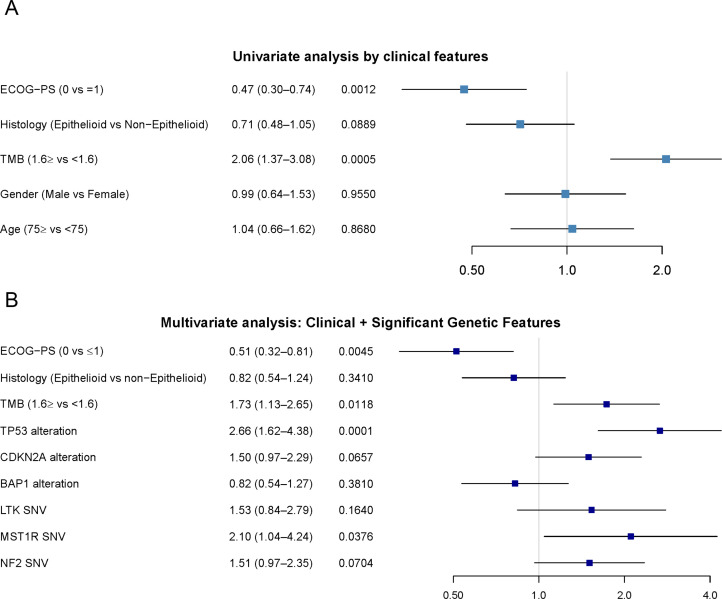
Univariate analysis and multivariate analysis focusing on clinical characteristics and genomic alternations. (A) Forest plot showing hazard ratios from univariate Cox regression analysis of overall survival, stratified by the clinical features. The x-axis indicates hazard ratios, with values >1 suggesting worse prognosis. (B) Forest plot showing hazard ratios from multivariate Cox regression analysis of overall survival, stratified by the clinical features and genomic changes. The x-axis indicates hazard ratios, with values >1 suggesting worse prognosis. Abbreviations: ICI, immune checkpoint inhibitor; TMB, tumor mutation burden; ECOG-PS, Eastern Cooperative Oncology Group Performance Status; SNV, single nucleotide variant.

Although the number of patients was limited, exploratory analyses were performed to investigate the relationship between genomic alterations and prognosis. In univariate analyses based on single nucleotide variants (SNVs), mutations in *TP53, LTK, SMAD4, CDKN2A, BRAF*, and *MST1R* were associated with shorter OS (Fig. 4C). Univariate analyses incorporating clinical characteristics and TMB showed that patients with PS 0 had significantly better survival, and a trend toward improved OS was observed in patients with epithelioid histology (Fig. 5A). No significant differences were found according to age (cutoff, 75 years) or sex. Subsequently, multivariate analysis was conducted by integrating clinical and genomic variables. Gene alterations with a prevalence of less than 10% were excluded from this model. The results indicated that PS 0 was a favorable prognostic factor, whereas high TMB, *TP53, MST1R* and *CDKN2A* alterations were associated with poor prognosis (Fig. 5B).

Collectively, these findings suggest that combining clinical characteristics with genomic alterations may provide a useful framework for predicting outcomes in patients with PM.

## Discussion

In this study, we elucidated the genetic alterations associated with PM in Japanese patients by analyzing a large-scale clinical-genomic database, C-CAT. This represents one of the largest cohorts to date in which genomic data on PM have been integrated with detailed clinical information. The most frequently observed gene mutations in Japanese pleural mesothelioma were *BAP1, NF2, NOTCH3, TP53*, and *STK11*. In terms of copy number alterations, losses of *CDKN2A, CDKN2B, MTAP, BAP1,* and *TEK* were most common. The median TMB was 1.26, which was largely consistent with previously reported data from other cohorts, including those from TCGA. Given the scarcity of genomic data on PM in Asian populations, our study provides important insights into the molecular landscape of this disease in Japan. Furthermore, comparative analyses with other datasets, such as TCGA, systematically revealed both similarities and differences in the genetic profiles of PM between Japanese patients and those from other populations.

According to the World Health Organization (WHO) classification (5th edition), PM is histologically classified into three major subtypes: epithelioid, sarcomatoid, and biphasic mesothelioma. These histologic subtypes are clinically important as they form the basis for therapeutic decision-making [16], for example, in the case of surgical treatment, patients with the sarcomatoid subtype generally have a poor prognosis, and surgery is not recommended for this histologic type [17]. In systemic chemotherapy, a combination of platinum agents and pemetrexed has shown greater efficacy in patients with the epithelioid subtype of PM. Although chemotherapy is considered to provide superior benefit in epithelioid PM in the present study, clinical evidence indicates that immunotherapy-based regimens may offer comparable efficacy in this histologic subtype [18]. In contrast, regimens containing ICIs such as IPI/NIVO or platinum–pemetrexed combined with pembrolizumab have been reported to provide additional therapeutic benefits and higher efficacy in patients with non-epithelioid PM [2,4]. Consequently, systemic therapy selection remains influenced by treatment availability and multidisciplinary/shared decision-making when more than one therapeutic option is accessible. As described, clinical treatment strategies for PM are determined based on the current WHO classification, making histopathological subtyping a crucial factor in therapeutic decision-making. However, recent reports integrating large-scale whole-genome sequencing data with transcriptomic and epigenomic analyses using multiomics approaches have suggested that the current WHO classification explains only approximately 10% of the molecular-level heterogeneity observed among patients [19]. These findings underscore the importance of incorporating clinical and genomic information in addition to histopathological classification to achieve a more comprehensive understanding and accurate stratification of PM.

Previous studies on genetic alterations in PM have reported that mutations or deletions in *BAP1, NF2, CDKN2A*, and *MTAP* are among the most frequently observed genomic abnormalities [5,6,20]. A recent study examining the relationship between genomic alterations and the efficacy of IPI/NIVO therapy reported that patients with *MTAP* or *CDKN2A/B* loss exhibited shorter OS than those with wild-type tumors, whereas patients with *BAP1* loss showed improved OS. In contrast, no significant difference in OS was observed between *MTAP* loss and wild-type patients [21]. In the present study, a similar trend was observed in the univariate analysis. *MTAP* and *CDKN2A/B* are located in the chromosomal region of 9p21, and the loss of 9p21 has been reported in other cancer types to be associated with reduced tumor-infiltrating lymphocytes and diminished responsiveness to ICIs [22]. In PM, for which ICIs are among the key therapeutic options, our results suggest that these genomic alterations may affect immune cell activation and could partly explain the variability in ICI responsiveness observed in the Japanese PM cohort.

In the present study, TMB and *TP53* expression were suggested as potential prognostic factors in Japanese patients with PM. *TP53* is a well-known tumor suppressor gene that plays a pivotal role in cell cycle arrest, apoptosis, senescence, DNA repair, and metabolic regulation, and its association with various cancers has been extensively documented [23]. A systematic review evaluating prognostic factors across multiple cancer types identified *TP53* alterations as an indicator of poor prognosis in various malignancies, including breast, colorectal, lung, renal, and endometrial cancers, as well as leukemia, suggesting its pan-cancer relevance as a negative prognostic factor [24]. Although these reports were published before the clinical introduction of ICIs, studies on PM have suggested that patients harboring *TP53* alterations may have a poorer prognoses [25]. Our findings demonstrated that even after the clinical implementation of ICIs, TP53-positive patients in the Japanese PM cohort continued to show poorer survival outcomes.

TMB has been established as a predictive biomarker for ICI efficacy, and previous studies across multiple cancer types (excluding mesothelioma) have demonstrated that high TMB is generally associated with improved OS, although the magnitude of this association varies among different tumor types [14,26]. Because high TMB (≥10 mutations per megabase) has been associated with favorable responses to the ICI pembrolizumab, this agent has been approved by the U.S. Food and Drug Administration (FDA) and also in Japan, and is now available for clinical use [27]. Consistent with previous studies [21], our findings confirmed that PM typically harbors a low TMB. Using a cutoff value of 1.6, patients with a higher TMB had worse survival rates, whereas ICI-treated patients within this group demonstrated improved outcomes. However, given the generally low TMB observed in the PM, this result should be interpreted cautiously. In the present study, a total of 23 liquid biopsy specimens were submitted for analysis. Liquid biopsy represents a minimally invasive approach for detecting and analyzing tumor-derived components, including circulating tumor DNA (ctDNA), circulating tumor cells, and extracellular vesicles, from body fluids such as blood, thereby providing insights into the molecular characteristics and temporal dynamics of tumors [[28], [29], [30]]. Unlike conventional tissue biopsy, liquid biopsy enables longitudinal assessment and has been increasingly applied to the monitoring of therapeutic response, identification of resistance-associated molecular alterations, and evaluation of minimal residual disease [29,30]. In particular, ctDNA analysis has been shown to partially reflect spatial and temporal intratumoral heterogeneity, underscoring its potential utility in the advancement of precision oncology [28,30]. Nevertheless, several limitations remain, including reduced analytical sensitivity in patients with low tumor burden, lack of standardization across pre-analytical and analytical workflows, and challenges in the clinical interpretation of detected alterations, indicating that further validation through well-designed prospective studies is warranted before liquid biopsy can be fully incorporated into routine clinical decision-making [31].

This study had several limitations. This was a retrospective database analysis with potentially incomplete clinical data and delayed survival information updates. Due to its retrospective design, there is a risk of selection bias in determining which patients underwent CGP testing. Moreover, the CGP assays used in Japan are based on targeted sequencing and provide less genomic coverage than whole-exome or transcriptomic analyses, which may have contributed to differences from previous studies. In addition, information on surgical and radiation treatments was not available in the C-CAT database. We were unable to evaluate clonal hematopoiesis of indeterminate potential in the present analysis, and perform comprehensive gene co-alterations; therefore, our ability to evaluate the independent contribution of each mutation may have been limited. Furthermore, because of the relatively small sample size, analyses, such as propensity score matching, could not be performed.

In conclusion, we elucidated the genomic landscape of PM in Japanese patients, addressing the paucity of data in Asian populations. Although actionable genetic alterations that are directly targeted by existing drugs are limited, our findings suggest the potential of several factors as clinically relevant biomarkers. Further studies are warranted to facilitate the development of novel therapeutic agents that may improve the survival outcomes of patients with PM.

## Disclosure

### Funding information

None

### Ethics statement

− Approval of the research protocol by an Institutional Reviewer Board: This study was conducted in accordance with the Declaration of Helsinki and its later amendments or comparable ethical standards. This study was approved by the Institutional Reviewer Board of Nagasaki University Hospital (approval no. 25040305).
− Informed Consent: All informed consent was obtained from the patients.
− Registry and the Registration No. of the study/trial: N/A
− Animal Studies: N/A

### Data sharing and data accessibility

The data generated in this study are available upon request from the corresponding author.

## CRediT authorship contribution statement

**Hirokazu Taniguchi:** Conceptualization, Data curation, Formal analysis, Investigation, Methodology, Writing – original draft. **Kazumasa Akagi:** Conceptualization, Data curation, Formal analysis, Investigation, Methodology, Writing – original draft. **Takahito Fukuda:** Data curation, Formal analysis, Investigation, Methodology, Writing – review & editing. **Takuya Honda:** Investigation, Methodology, Writing – review & editing. **Hirokazu Kurohama:** Investigation, Methodology, Writing – review & editing. **Nozomi Ueki:** Investigation, Methodology, Writing – review & editing. **Yuki Matsuoka:** Investigation, Methodology, Writing – review & editing. **Emiko Udo:** Data curation, Investigation, Methodology, Writing – review & editing. **Saya Yahata:** Data curation, Investigation, Writing – review & editing, Methodology. **Shoko Miura:** Investigation, Methodology, Writing – review & editing. **Hiromi Tomono:** Writing – review & editing, Investigation. **Noritaka Honda:** Writing – review & editing, Investigation. **Yosuke Dotsu:** Investigation, Writing – review & editing. **Midori Matsuo:** Writing – review & editing, Investigation. **Shinnosuke Takemoto:** Investigation, Writing – review & editing. **Izumi Sato:** Formal analysis, Investigation, Writing – review & editing, Methodology. **Shinji Okano:** Methodology, Writing – review & editing. **Masahiro Nakashima:** Methodology, Writing – review & editing. **Hiroshi Mukae:** Investigation, Writing – review & editing. **Kazuto Ashizawa:** Investigation, Methodology, Writing – review & editing.

## Declaration of competing interest

The work described has not been published previously except in the form of a preprint, an abstract, a published lecture, academic thesis or registered report.

The article is not under consideration for publication elsewhere.

The article's publication is approved by all authors and tacitly or explicitly by the responsible authorities where the work was carried out.

H.Taniguchi has received speaking honoraria from 10.13039/100010795Chugai Pharmaceutical. T.H has received speaking honoraria from 10.13039/100010795Chugai Pharmaceutical, 10.13039/501100013170Ono Pharmaceutical, and 10.13039/100001009Bristol Myers Squibb. S.T has received speaking honoraria from 10.13039/100010795Chugai Pharmaceutical, 10.13039/501100013170Ono Pharmaceutical, and 10.13039/100001009Bristol Myers Squibb. H.M has received speaking honoraria from 10.13039/100010795Chugai Pharmaceutical and 10.13039/100001009Bristol Myers Squibb. The other authors declare no conflicts of interest for this work.

## Appendix Supplementary materials

Supplementary Fig. S1. Oncoplot displaying the top 50 most frequently mutated genes based on single nucleotide variants in the TCGA-MESO project of The Cancer Genome Atlas. Supplementary Fig. S2. Kaplan–Meier curves comparing overall survival among patients with epithelioid, sarcomatoid, biphasic and unclassified histology. Supplementary Fig. S3. (A) Comparison of TMB across types of CGP. P value was calculated by one way ANOVA test and Tukey's multiple comparisons test. *P<0.05, ****P<0.0001, ns; not significance (B) Kaplan–Meier curves comparing overall survival between patients with high and low TMB in liquid biopsy based CGP (left) and tissue based CGP (right). Abbreviations: TMB, tumor mutation burden. Supplementary Fig. S4. Kaplan–Meier curves comparing overall survival between patients with CDKN2A and/or MTAP loss. Abbreviations: CNA, copy number alteration. Image, application 1

Supplementary Table S1. List of alterations of CGP panels Image, application 2
