# Supplementary material for: Genomic landscape of pleural mesothelioma in Japanese patients: A comprehensive analysis using nationwide database
Source: Transl Oncol. 2026 Mar 18;67:102731. doi: 10.1016/j.tranon.2026.102731 (PMC13018873; doi:10.1016/j.tranon.2026.102731)

Suppprementary Figure 1

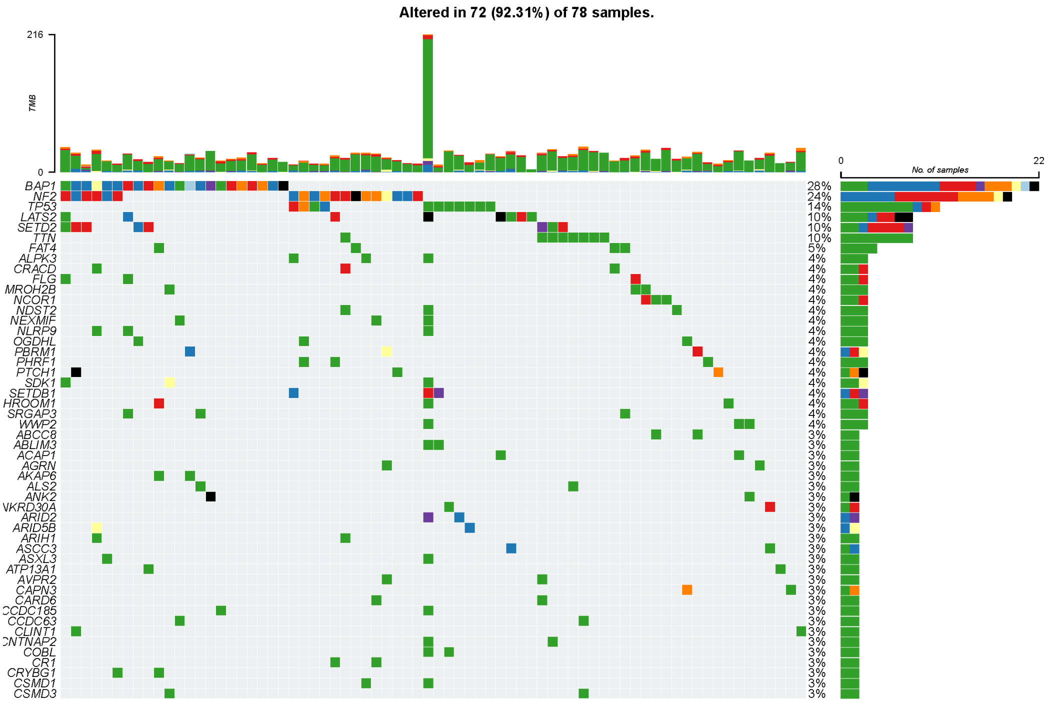

Missense_Mutation
Splice_Site
Frame_Shift_Del
In_Frame_Del
Nonsense_Mutation
Nonstop_Mutation
Frame_Shift_Ins
Multi_Hit

Suppremetary Figure 2

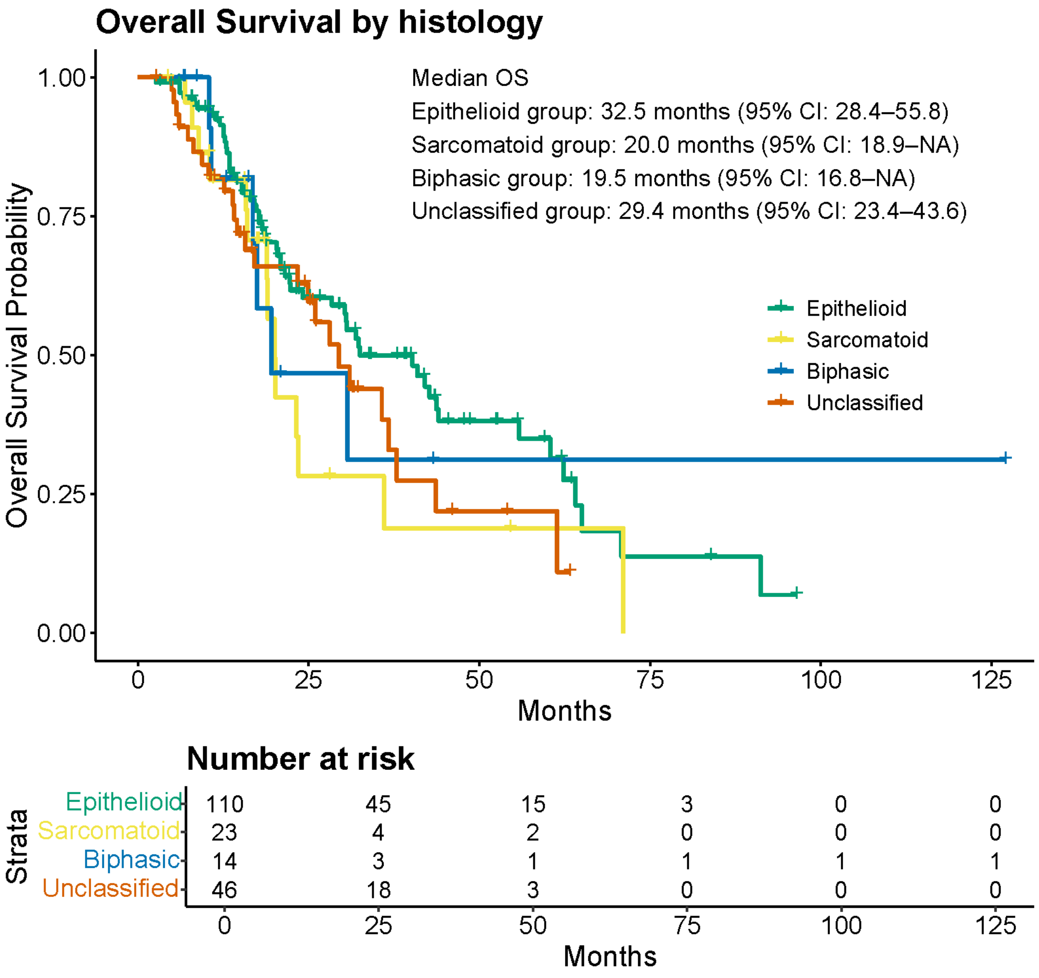

Supprementary Figure 3

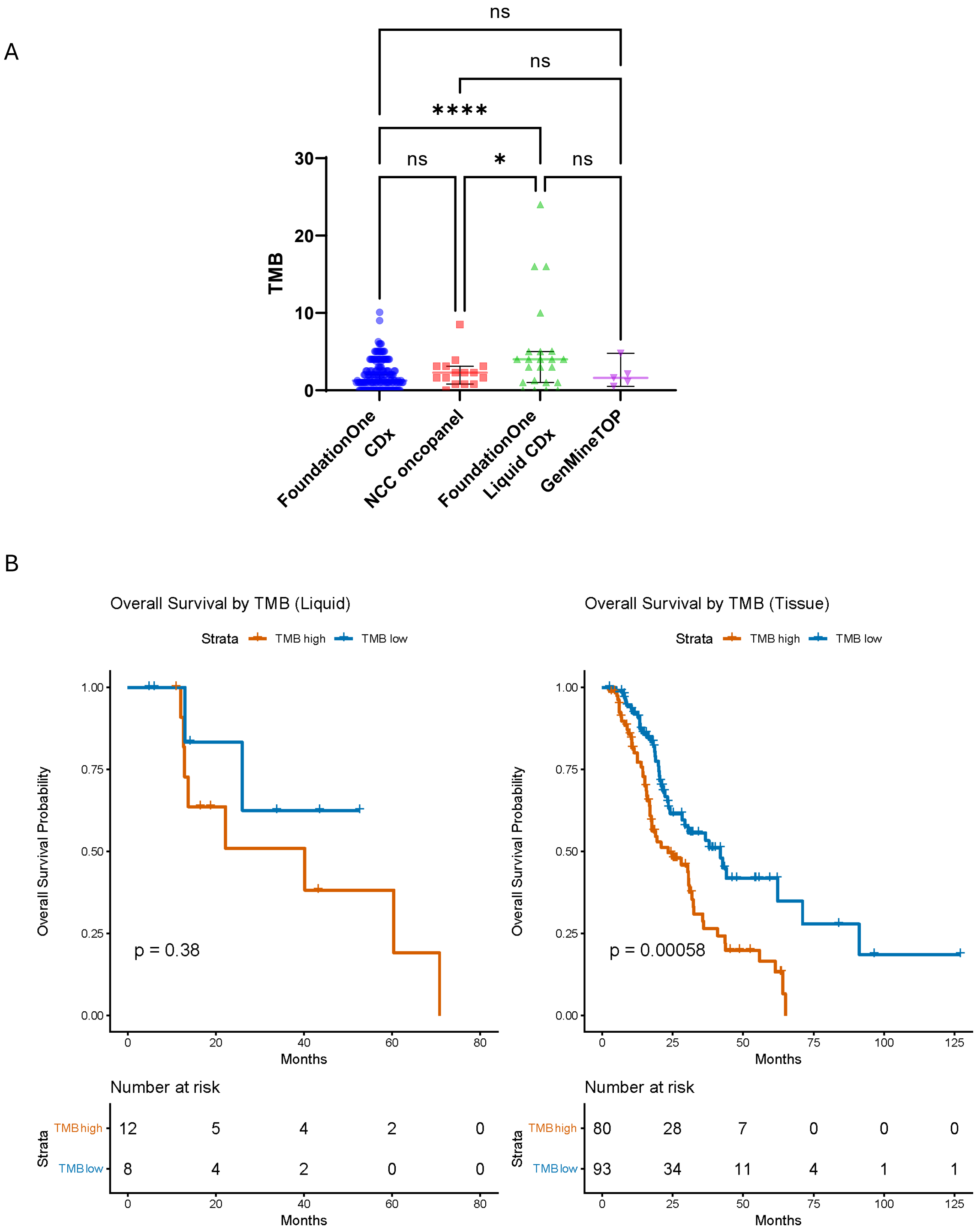

Supprementary Figure 4

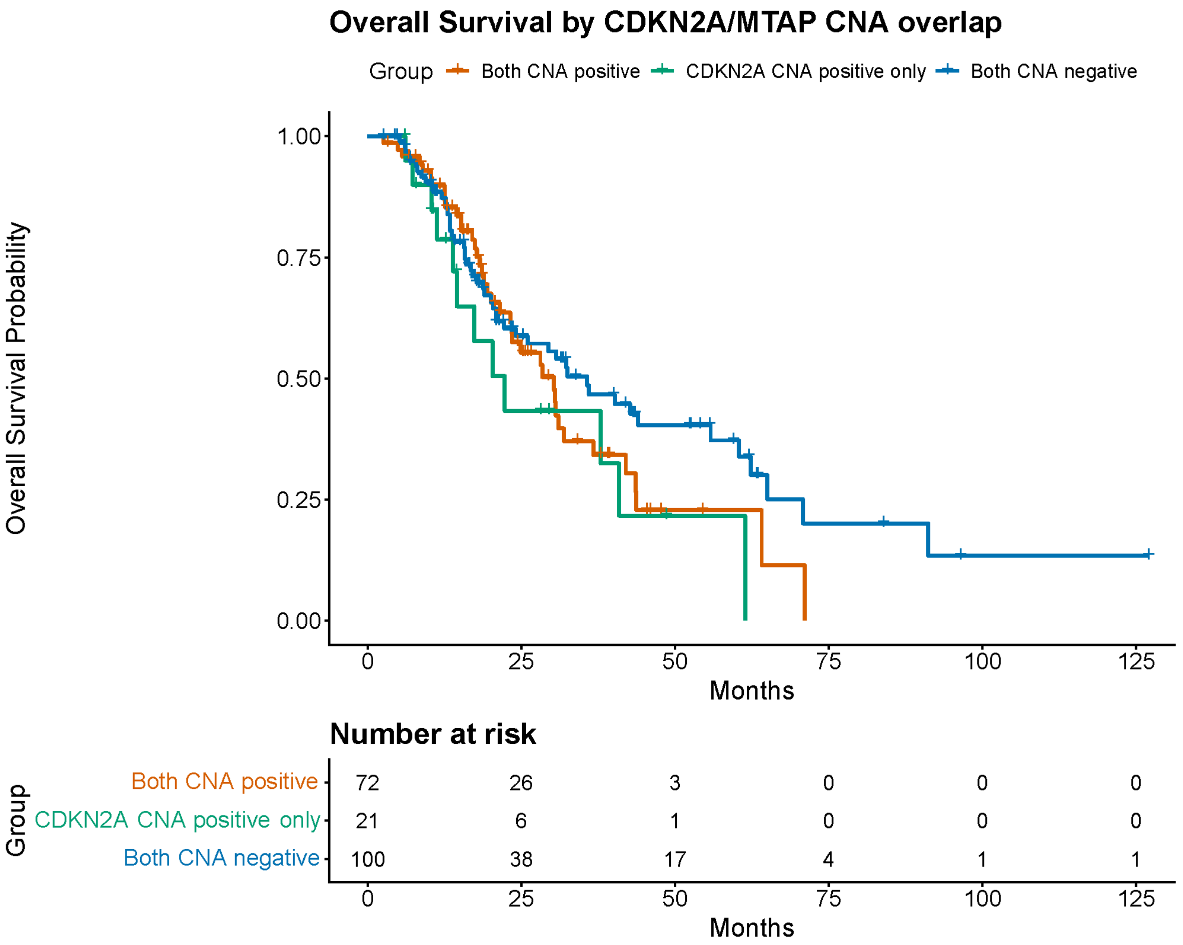

Supplement: Supplementary file 1 — Supplementary Fig. S1. Oncoplot displaying the top 50 most frequently mutated genes based on single nucleotide variants in the TCGA-MESO project of The Cancer Genome Atlas. Supplementary Fig. S2. Kaplan–Meier curves comparing overall survival among patients with epithelioid, sarcomatoid, biphasic and unclassified histology. Supplementary Fig. S3. (A) Comparison of TMB across types of CGP. P value was calculated by one way ANOVA test and Tukey's multiple comparisons test. *P<0.05, ****P<0.0001, ns; not significance (B) Kaplan–Meier curves comparing overall survival between patients with high and low TMB in liquid biopsy based CGP (left) and tissue based CGP (right). Abbreviations: TMB, tumor mutation burden. Supplementary Fig. S4. Kaplan–Meier curves comparing overall survival between patients with CDKN2A and/or MTAP loss. Abbreviations: CNA, copy number alteration. [file mmc1.pdf]
